# From Fragment to One Piece: A Review on AI-Driven Graphic Design

**DOI:** 10.3390/jimaging11090289

**Published:** 2025-08-25

**Authors:** Xingxing Zou, Wen Zhang, Nanxuan Zhao

**Affiliations:** 1 School of Fashion and Textiles, The Hong Kong Polytechnic University, Hong Kong SAR 999077, China; 2 School of Computing, Informatics, and Decision Systems Engineering, Arizona State University, Tempe, AZ 85281, USA; wenzhang.ccm@gmail.com; 3 Adobe Inc., San Jose, CA 95110-2704, USA; nanxuanzhao@gmail.com

**Keywords:** AI in graphic design, design interpretation, creative process

## Abstract

This survey offers a comprehensive overview of advancements in Artificial Intelligence in Graphic Design (AIGD), with a focus on the integration of AI techniques to enhance design interpretation and creative processes. The field is categorized into two primary directions: perception tasks, which involve understanding and analyzing design elements, and generation tasks, which focus on creating new design elements and layouts. The methodology emphasizes the exploration of various subtasks including the perception and generation of visual elements, aesthetic and semantic understanding, and layout analysis and generation. The survey also highlights the role of large language models and multimodal approaches in bridging the gap between localized visual features and global design intent. Despite significant progress, challenges persist in understanding human intent, ensuring interpretability, and maintaining control over multilayered compositions. This survey aims to serve as a guide for researchers, detailing the current state of AIGD and outlining potential future directions.

## 1. Introduction

Forecasts by McKinsey and PricewaterhouseCoopers suggest that generative AI in graphic design could potentially contribute more than USD 8 trillion to the global economy by 2030. Research interest in this area has continuously increased.

**Figure 1:**
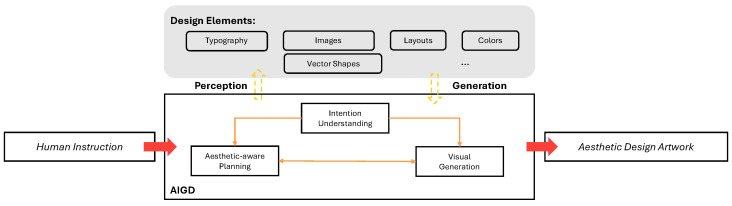
General pipeline of Artificial Intelligence in Graphic Design (AIGD).

The academic evolution of Artificial Intelligence in Graphic Design (AIGD) reveals two distinct phases. Early efforts focused on decomposing design tasks into atomic components—typography generation [1], layout optimization [2,3], and color palette recommendation [4,5,6,7,8]—employing specialized models for each subtask. While effective for generating individual elements, this decompositional approach introduced systemic fragmentation that persists in current research [9]. Recent breakthroughs in large-scale text models have catalyzed the evolution of generative visual models [10,11]. Within graphic design [12], as illustrated in Figure 1, this progress reflects a paradigm shift: from optimizing isolated elements (e.g., typography, images, vector shapes, layouts, and colors) to holistic creative systems capable of maintaining aesthetic consistency across entire design workflows—from human instruction to final artwork. Figure 1 serves as a visual representation of this paradigm shift, highlighting the core methodology and nature of the results achieved by contemporary AI-driven design systems. The figure is divided into three key stages: (1) Human Instruction and Intent Definition: The initial phase captures how users provide input, including textual descriptions, style preferences, and design objectives. This step defines the creative intent and sets the foundation for the AI system to interpret and act upon. (2) AI-Driven Component Optimization: The middle section of the figure illustrates how the system processes the input to generate and refine individual design elements. For example, the AI applies advanced algorithms to create typography, shapes, and layouts that align with the specified instructions. Tools like Adobe Firefly excel in this stage, leveraging machine learning to produce assets that are both visually appealing and contextually relevant. (3) Holistic Workflow Integration: The final stage presents the system’s ability to harmonize all design elements into a cohesive output. This includes maintaining aesthetic consistency across colors, proportions, and layouts, ensuring the final artwork adheres to the creative intent defined in the first stage. By following this structured workflow, as depicted in Figure 1, these systems demonstrate how they bridge the gap between isolated design tasks and fully integrated creative processes. This figure not only illustrates the methodology but also underscores the transformative nature of these AI-driven solutions, which enable designers to achieve faster, more efficient, and aesthetically consistent results. Tools like Adobe Firefly are emblematic of this trend, showcasing the potential of AI to redefine creative workflows.

As summarized in Table 1, recent surveys in graphic design have explored various dimensions of the field. Ref. [13] analyzes vector graphics through mathematical foundations and content creation stages; ref. [14] delves into layout generation aesthetics and technologies; ref. [9] provides a taxonomy of graphic design intelligence. Ref. [15] reviews graphic layout generation focusing on implementation and interactivity, while [16] investigates challenges and future functional needs for AI-generated image tools in graphic design through designer interviews. Table 1 below highlights the key contributions of these works in comparison to our survey, focusing on the unification of cognitive and generative tasks.

**Scope of the Reviews.** The multimodal era has seen numerous attempts at cross-modal integration through vision–language models [17,18]. While these efforts represent significant progress, they have largely struggled to bridge the semantic gap between localized visual features and global design intent [19]. Recent strides in LLM-driven design systems [19,20] mark a pivotal shift by introducing a convergence path where generative processes are guided by explicit design rationale encoded in latent spaces [21,22]. Building on these advances, this article introduces a novel perspective on AI-Driven Graphic Design (AIGD), emphasizing design understanding and creativity as central themes. Unlike prior studies that narrowly focused on isolated technical improvements, our work takes a holistic approach to the design process. Specifically, we bridge the gap between abstract concepts and tangible creations, offering a comprehensive framework that enables readers to grasp the full scope of AIGD. Moreover, we demonstrate how the latest AI advancements can not only streamline workflows but also significantly enhance the creative potential of graphic design. By reframing AIGD in this way, we provide a fresh lens that highlights the transformative potential of AI in fostering creativity across the design spectrum.

**Figure 2:**
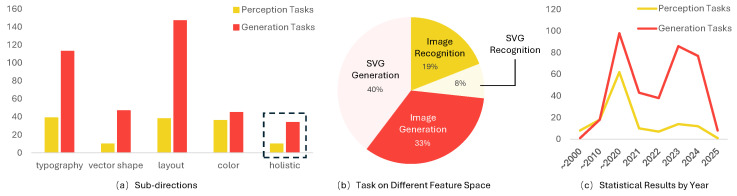
Overview of research publications in AIGD: (**a**) subdirections, (**b**) task on different feature space, and (**c**) statistical results by years. Holistic indicates systems capable of processing multiple or all design elements.

To address the evolving landscape of AIGD, this survey conducts an extensive and in-depth analysis. By examining AIGD research through the dual lenses of design semantics—such as visual hierarchy, typography, and color theory—and creative workflows—including ideation, iteration, and refinement—we establish a unified framework to assess advancements in this domain. This approach contrasts with previous analyses, which were often fragmented, by providing a structured examination of how AI models both interpret and generate meaningful design artifacts, such as raster and vector graphics, while maintaining artistic intent and adaptability. We conducted a systematic search across major academic databases, including IEEE Xplore, ACM Digital Library, Scopus, Web of Science, using keywords such as “graphic design”, “AI in design”, “layout generation”, and “generative design”. From the initial pool of papers from 2000 to 2025, we applied inclusion and exclusion criteria based on relevance to AI-driven graphic design, leading to a final dataset of 500 papers. Papers were categorized into key themes based on their primary focus, including (i) cognitive tasks (e.g., reasoning, decision-making), (ii) generative tasks (e.g., image and layout generation), and (iii) hybrid approaches integrating both. This categorization was performed by two independent researchers to minimize bias, with disagreements resolved through discussion. Within these categories, we delved into four key subtasks associated with design elements: non-text objects, text characters, aesthetic elements, and layout. The statistical distribution between cognitive and generative research is visually summarized in Figure 2. Our findings highlight several trends:

(1) Recent research predominantly focuses on individual subtasks, with studies considering graphic design as a holistic endeavor emerging prominently since 2023. (2) While raster images are generally more common in AI research, vector images are particularly significant in AIGD due to attributes like lossless scaling. (3) Tasks such as font and layout generation are more frequently addressed for raster images, while vector-based studies tend to focus on non-text objects. This discrepancy is often due to the broader research on raster image generation not specifically targeting graphic design, resulting in domain incompatibility. (4) There is a marked increase in enthusiasm for AIGD, with significant growth in interest for generation tasks since 2010, especially noted from 2022 onwards.

This survey aims to provide a comprehensive review of the methodologies involved in these subtasks, discuss ongoing challenges, unresolved issues, and suggest directions for future research. The remainder of this article is structured as follows: Section 2 presents the background of graphic design, introducing relevant concepts and involved subtasks. Section 3 and  Section 4 discuss cognitive and generative tasks, respectively. We focus on research strictly related to graphic design or closely associated domains. Given the extensive scope of this survey, we offer condensed information to enhance understanding and outline research branches. Finally, in Section 5, we discuss the state of the art in the era of multimodal large language models, existing challenges, and potential future trends, considering graphic design from a holistic rather than a piecemeal approach.

## 2. Background

Graphic design aims to deliver information clearly while presenting it in an appealing visual way [23]. It involves design elements, including non-text objects (images and vector shapes) and text characters (typography), to create aesthetic narratives [23] through visual harmony layout and aesthetic elements, particularly colors. Graphic design typically employs two principal data types: raster and vector images.

- A raster image is a two-dimensional array storing pixel values, with the pixel as its fundamental unit influenced by resolution.
- Scalable Vector Graphics (SVG) uses mathematical descriptions to record content, such as parameters to draw straight lines.

We first define the problem of AIGD under a unified mathematical formulation. Let E=T∪O represent the set of all design elements, where *T* is the set of text elements (typography), and *O* is the set of non-text elements (images or vector shapes). Each element e∈E in the design is described by two kinds of features: (1) its attribute vector $a_{e}$, which may include style, size, color, etc., and (2) its design content $c_{e}$, such as visual context of objects. Therefore, a candidate design artifact is a set of element features, $D(A=\cup a_{e},C=\cup c_{e})$. The objective of the design is to find an optimal D(A,C) which maximize the following function:

(1) $$D\left(A,C\right)=\arg\max_{A,C}V\left(L\left(A\right),C|I\right)$$

where V(.) measures the aesthetic value of design *D* under the constraint of user intention *I*. L(A) is the layout of design elements,

(2) $$L\left(A\right)=h\left(\tilde{A}|C\right)\geq \tau$$

which follows the certain design principles (h(.)) providing best harmonic settings among design objects. τ is the minimal harmonic score. In Equation (2), not all attributes are applicable to the layout. Typically, a subset $\tilde{A}=\left(p_{i},s_{i},\theta_{i}\right)$ represents the core parameters, where $p_{i}\in R^{2}$ represents the position coordinates of element $e_{i}$ (for a two-dimensional layout), $s_{i}\in R^{+}$ represents the size of an element $e_{i}$ (e.g., width and height), and $\theta_{i}\in S$ represents other states of element $e_{i}$ (e.g., rotation angle, transpose, etc.). Considering the human centric nature of graphic design, human intention *I* steers the exploration of D(A,C). However, interpretation of human intention is complex and requires consideration of individual preference. It is provided as a part of system inputs, in terms of multimodal instructions, reference images, or templates.

Ideally, it is anticipated that Equation (1) could be resolved within a unified pipeline. The major components would be capable of comprehending design intentions *I*, acquiring basic relevant elements $E_{basic}$, orchestrating graphic layouts L(A), and ensuring the visual harmony of the produced outcomes V(D). Recent studies published between 2023 and 2024 have explored the potential of LLMs in graphic design, as evidenced by works such as those by Dou et al. (2024) [24], Huang et al. (2024) [25], and others [19,20,26,27]. However, these efforts are still in the early stages and have yet to achieve a deep understanding and professional generation.

The primary goal of graphic design is to deliver information. While visual elements, particularly images and vector shapes, enhance aesthetic appeal, text elements directly communicate the design’s theme and play a more central role in information delivery. This relative importance is reflected in statistical data from the field, indicating that 31% of tasks are text-related, while only 12% focus on non-text elements, including tasks that involve both types of content. Text in graphic design encompasses various attributes such as fonts, glyphs, artistic styles, and semantic typography, all crucial for effective communication. Another significant factor is the meticulous arrangement of these elements. Key messages in the text should be highlighted, and design considerations like adequate white space, optimal contrast, and visual balance are essential for a well-managed layout. Research specifically targeting these considerations accounts for more than 38% of studies in this area. Additionally, achieving aesthetic appeal through techniques like color harmony is also critical, representing 13% of research interests. Due to the complex nature of these tasks, much of the existing research has been divided into distinct sub-directions, primarily categorized into two areas: perception tasks [28,29,30,31,32,33,34,35] and generation tasks [36,37,38,39,40,41,42,43,44,45,46,47].

## 3. Perception Tasks

Understanding design intent is the first step towards AIGD, which requires a model with a basic knowledge of graphic design principles. Therefore, this section presents the methodology evolution separately for each subtask, including non-text element perception in Section 3.1, text element perception in Section 3.2, layout analysis in Section 3.3, and aesthetic understanding in Section 3.4. Two main types of data are primarily used in graphic design.

### 3.1. Non-Text Element Perception

#### 3.1.1. Object Recognition in Raster Image

Numerous comprehensive surveys and reviews have documented advances in non-text object recognition [48]. Building upon the areas not extensively covered by these surveys, recent progress in multimodal large language models (MLLMs) [49] has significantly expanded the capabilities of LLMs to process and interpret text and visual data. These MLLMs have demonstrated remarkable proficiency in vision–language tasks, such as image captioning and visual question answering. Furthermore, contemporary research has begun to explore the potential of using textual output from LLMs to steer external vision expert models to perform a variety of vision-centric tasks [50]. In object detection, such expert models include systems such as DETR [51], which are designed to improve the accuracy and efficiency of detecting and interpreting visual objects.

#### 3.1.2. SVG Recognition

Traditional methods employ rule-based graph-matching techniques, such as visibility graphs [52] and attributed relational graphs [53]. YOLaT [54] first proposed a learning-based method that represented vector graphics using graphs based on the Bézier curve, where object detection was conducted based on the predictions of a Graph Neural Network (GNN). However, this work modeled only in a flat GNN architecture with vertices as nodes, ignoring the higher-level information of vector data. The follow-up work by [24], YOLaT++, learns multi-level abstraction features from primitive shapes to curves and points. They also provide a new dataset for chart-based vector graphics detection and chart understanding, which includes vector graphics, raster graphics, annotations, and raw data for creating these vector charts.

### 3.2. Text Element Perception

#### 3.2.1. Optical Character Recognition (OCR)

To facilitate text recognition, it is essential to locate the text area within an image [55]. Popular text detection algorithms can be broadly categorized into regression-based, segmentation-based, and detection transformers. Regression-based algorithms draw from general object detection methods, which treat text detection as a unique scenario within target detection, such as TextBoxes [56] (based on the Single Shot Multi-box Detector (SSD) [57]) and CTPN [58] (based on Faster R-CNN [59]), among others. ABCNet [60] is the first to introduce Bézier curve control points for arbitrary-shaped texts. On the other hand, segmentation-based algorithms, inspired by Mask R-CNN [61], have significantly improved text detection across various scenes and shapes but entail complex post-processing, speed issues, and challenges in detecting overlapping text. DETR [51] represents a pioneering model introducing a fully end-to-end transformer-based paradigm. However, DETR’s training convergence and feature resolution limitations have hindered its competitiveness compared to traditional detectors. Other variants include Conditional-DETR [62] and Anchor-DETR [63]. Furthermore, approaches like DN-DETR [64] and MaskDINO [65] concentrate on label assignment strategies, significantly improving matching stability.

Once text is detected, text recognition algorithms identify the content within the detected areas. It is typically divided into regular and irregular text recognition based on the shape of the text. Regular text includes printed fonts and scanned text, whereas irregular text often appears non-horizontal and may exhibit bending, occlusion, and blurring. Historically, the mainstream approach involved segmentation and single-unit recognition, utilizing connected domain analysis to identify potential text segmentation points. Post-2016, the focus shifted to text line recognition. Early works using DNNs as feature extractors for scene text recognition include [66]. However, many texts in natural scenes have arbitrary shapes and layouts, making it difficult to transform them into horizontal texts through the proposed interpolation methods. To this end, the later studies focus on identifying granular-level elements, such as characters, and semantically encoding their relationships to enhance the recognition of irregular text [67,68,69]. Many recent works have introduced a growing trend of generative models into scene text recognition. Ref. [70] proposed transforming the entire scene text image into corresponding horizontally written canonical glyphs to promote feature learning. Through their experiments, the guidance of canonical glyph forms proved effective for feature learning in STR.

#### 3.2.2. Font Recognition

Fonts are typically designed with unique characteristics, such as stroke width, serifs, aspect ratio, spacing or slant/italicization [71]. Early font recognition works attempted to recognize fonts via these artificial font features [72,73]. While artificial font features worked reasonably well in controlled scenarios, they faced several challenges: (1.) Font variability: Fonts with subtle differences in design could be difficult to differentiate using simple features. (2.) Noise in input data: Scanned documents or degraded images introduced noise that could distort features like stroke width or spacing. (3.) Handwritten vs. printed fonts: Artificial features were less effective for recognizing handwritten or highly stylized fonts. (4.) Limited scalability: Adding new fonts required manually defining additional rules or features. As the learning-based method became popular, Wang et al. built a Convolutional Neural Network with domain adaptation techniques for font recognition, applying deep neural networks to font recognition for the first time [74]. This method was followed by that of Bharath et al., who utilized SVM for English font recognition, focusing on character image distances [75]. The research was further expanded by Liu et al., who introduced a multi-task adversarial network for Japanese fonts, employing a GAN to preprocess scene text images prior to recognition [76]. The introduction of the FontCLIP latent space further expands the possibilities for font selection using out-of-domain attributes and scripts, improving flexibility [77].

### 3.3. Layout Analysis

**Figure 3:**
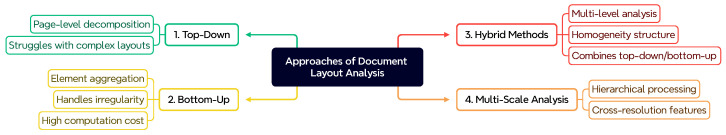
Overview of methods for layout analysis task.

Layout is composed of visual elements, typically characterized by properties such as type and position. As shown in Figure 3, traditional approaches use hand-crafted features to represent layout. For instance, Stoffel et al. designed features related to position, spacing, and font styles for document structure analysis [78]. Some methods employ neural networks, such as transformer [79] and Faster-RCNN [59], to encode layouts into low-dimensional continuous representations, showing promising results [80].

The layout analysis of graphic design shares the common foundation of other aesthetic-aware layout analyses, e.g., Document Layout Analysis (DLA), where domain knowledge can be easily transferred to all general graphic design. DLA methodologies include bottom-up, top-down, hybrid, and multi-scale approaches. The top-down approach starts with each page as a single large block, which is then subdivided into smaller sections, but struggles with complex layouts [81]. The bottom-up approach, starting at the granular level and aggregating adjacent elements into larger blocks, handles irregular layouts well but can be computationally demanding [82,83]. Hybrid methods combine these approaches and utilize a multi-level, homogeneity structure to improve layout analysis [84]. The adoption of CNNs has shifted DLA towards models that extract features directly from document pixels, addressing shortcomings of traditional methods. Early CNN models focused on textural features for segmenting segments but were less effective with elements like tables [85]. Recent developments like Gruning’s ARU-Net and Xu et al.’s multi-task FCN improve text line segmentation and contour detection [86,87]. These innovations emphasize the integration of semantic interpretation in DLA, highlighting the importance of understanding semantic relationships between document components. Ref. [88] highlights the importance of contextual relevance in element placement.

### 3.4. Aesthetic Understanding

The field of aesthetic understanding in graphic design has evolved from manual feature engineering to AI-driven multimodal systems [89]. Early approaches relied on handcrafted color metrics and rule-based harmony models, while modern methods leverage deep learning (GANs, VAEs, transformers) for context-aware palette generation and personalized recommendations. Concurrently, aesthetic assessment has transitioned from spatial/statistical analysis to neural architectures that model emotional impact and user preferences. This paradigm shift enables unified systems addressing both functional requirements (color discrimination) and affective dimensions (emotional resonance) across infographics, marketing materials, and interactive interfaces.

#### 3.4.1. Color Palettes Recommendation

Initial color recommendation systems relied heavily on manual feature extraction and regression models. For instance, Color Sommelier [90] introduced a harmony rating algorithm based on community-generated palettes, allowing users to iteratively select harmonious color schemes. However, these methods often overlooked the semantic meanings of colors and incorporated less critical features, leading to suboptimal predictions. The advent of deep learning marked a significant paradigm shift in color recommendation systems. Neural networks began to learn color feature representations from image color histograms, classifying images according to predefined categories. Early efforts included the use of neural networks on predefined color palettes tailored for specific themes, such as magazine cover design [91,92,93,94].

Recent studies have focused on recommending color palettes for information visualizations and statistical graphics, such as scatterplots and bar charts [95,96]. Beyond simple infographics, researchers have explored color palette recommendations for more complex visual designs, such as advertising posters and magazine covers. Yuan et al. [96] implemented a Variational AutoEncoder with Arbitrary Conditioning (VAEAC) to dynamically suggest colors for various infographic elements. The latest research in color recommendation increasingly focuses on generative models and region-specific recommendations. Refs. [97,98] developed a transformer-based masked color model for specific regions on landing pages and vector graphic documents. Ref. [99] utilized maximum likelihood estimation and conditional variational autoencoders within a transformer framework to recommend text and background colors for e-commerce mobile web pages.

#### 3.4.2. Other Aesthetic Attributes

Aesthetic visual quality assessment advances from traditional handcrafted feature extraction methods to sophisticated deep-learning approaches, with the trend from standardized assessment to more diverse and personalized evaluation. Early aesthetic visual quality assessment methods focused on extracting handcrafted features from images [100,101]. The introduction of models that incorporated human-describable attributes marked a significant advancement. Ref. [102] introduced a model that connected technical analysis with human perceptions, including elements such as composition, illumination, and content. Obrador et al. [103] evaluated photographs based on features like simplicity and visual balance. Ref. [104] developed models to predict users’ first aesthetic impressions of websites, based on visual complexity and colorfulness. With the advent of deep learning, the field underwent a transformative change. Lu et al. [105,106] utilized dual-column CNNs and a Deep Multi-Patch Aggregation Network (DMA-Net) to encode global image layouts better, significantly advancing the classification and understanding of aesthetic qualities. Recent studies have focused on personalized image aesthetics, exploring how users’ social behaviors and personal perceptions influence their aesthetic judgments. Cui et al. [107,108] addressed user-centric aesthetic assessment analysis. Chen et al. [109] introduced the Adaptive Fractional Dilated Convolution to maintain the original aspect ratios and composition of images.

### 3.5. Summary

The evolution of the visual cognition framework is illustrated by the transition from traditional methods to the adoption of deep learning techniques such as CNNs, subsequently incorporating GANs, transformers, and currently, LLMs. Similarly, although each subtask within AIGD progresses independently, their overall development trajectories align consistently with this. Another notable trend is research on vector images, which remains relatively sparse compared to raster images. Most studies on vector cognition have focused on SVG recognition, representing earlier efforts in the field. However, recent statistics indicate a growing interest in vector image research. This surge is attributed to the nature of vector representations, which are highly conducive to integration with LLMs for enhanced understanding and reasoning.

In text recognition, the main challenges addressed are OCR and font recognition. General approaches include techniques such as Faster R-CNN, text line or single character segmentation, and the input ranges from handwritten notes to natural scene images. The recognition process faces several challenges, including text distortion due to perspective changes, small text scale, stylized fonts, various font sizes and styles, decorative elements, multilingual text, image blur, and poor lighting conditions. Font recognition is another crucial aspect of text-related cognition and plays an essential role in graphic design where font styles are vital. However, the diversity of font styles poses a significant challenge to creating a comprehensive dataset, including unique styles such as italics and bold. This makes it difficult for models to learn to recognize diverse fonts.

In addition to text elements, layout analysis, especially in document structure, has received increasing attention from researchers. This analysis is a precursor to OCR, classifying and recognizing different elements in a document, such as text, images, tables, and titles. Recent research has made significant progress through large-scale language model-based tools such as LayoutLM, UDOP, and LiLT, which leverage multimodal transformer encoders pre-trained and fine-tuned for specific applications. Finally, aesthetic research has primarily focused on color matching, with additional analysis based on personality, photographic content, and direct visual feature computation. The subjective nature of aesthetics and the lack of clear principles or standards make it a challenging research area that lacks a strong framework or benchmarking system.

## 4. Generation Tasks

Graphic design needs elements with separate transparent backgrounds. Thus, we focus primarily on vector shape generation and the vectorization of artistic imagery. For ease of discussion, we categorize text element generation into the generation of text itself and the rendering of text within a scene. Meanwhile, we introduce works in layout generation and layout-based image generation. Finally, we address research focused on aesthetic refinement.

### 4.1. Non-Text Element Generation

#### 4.1.1. SVG Generation

SVG can be encoded as the sequence of 2D points connected by parametric curves, making the seq2seq model straightforward as an encoder/decoder basis [110,111,112,113,114]. SketchRNN [111] was a pioneer in employing LSTM-based VAEs for learning to draw strokes, representing sketches as sequences of pen positions and states. SVG-VAE [112] involves a two-stage training process that begins with an image-based VAE, followed by training a decoder to predict vector parameters from the latent variables. BézierSketch [115] focuses on generating Bézier curves, offering enhanced control over graphical forms of sketches. DeepSVG [110], a hierarchical autoencoder designed to learn representations of vector paths, contributes to the structural complexity of vector graphics. These methods heavily rely on datasets in vector form, which limits their generalization capabilities and their capacity to synthesize complex vector graphics. IconShop trains a BERT model for text-conditioned SVG generation of icons but is restricted to using paths [114].

Instead of directly learning an SVG, another method of vector synthesis optimizes towards a paired raster image during training. Ref. [116] observed vector graphics rasterization was differentiable after pixel prefiltering. Conditioned on this finding, the authors introduced a differentiable rasterizer that offered two prefiltering options: an analytical prefiltering technique and a multisampling anti-aliasing technique. The analytical variant was faster but could suffer from artefacts such as conflation. The multisampling variant was still efficient and could render high-quality images while computing unbiased gradients for each pixel with respect to curve parameters. That work enabled the supervision of the SVG generation under the guidance of a raster image. In other words, different from image generation methods that traditionally operate over vector graphics and require a vector-based dataset, recent work has demonstrated the use of differentiable renderers to overcome this limitation [117,118,119,120,121,122]. CanvasVAE defines vector graphic documents through a multimodal set of attributes, using variational autoencoders to integrate diverse graphical components [123]. Im2Vec is a method that employs a differentiable rasterization pipeline to generate complex vector graphics from raster training images [118]. Furthermore, recent advances in visual text embedding and contrastive language–image pre-training models have enabled a number of successful methods for synthesizing sketches, including CLIPDraw and CLIPasso [124,125]. In addition to using CLIP distance, VectorFusion [126] and DiffSketcher [122] combine a differentiable renderer with a text-to-image diffusion model for vector graphics generation. This type of method utilizes Score Distillation Sampling loss based on a text-to-image (T2I) diffusion model for optimizing SVG to align with text prompts across various applications such as fonts, vector graphics, and sketches [127,128,129]. However, due to the lack of geometric constraints, they often lead to path intersections or jagged effects. By adding geometric constraints to a Text-to-Vector (T2V) generation pipeline that optimizes local neural path representation, high-quality SVG graphics generation is achieved [130].

#### 4.1.2. Vectorization of Artist-Generated Imagery

Image vectorization is another alternative way to directly obtain the bitmap from imagery. Traditional vectorization techniques primarily depend on segmentation or edge detection to group pixels into larger regions, subsequently fitting vector curves and region primitives to these segments [131,132]. Challenges include aligning patch boundaries and automating mesh generation [132,133]. In contour-based vectorization, simpler elements such as lines, circles, and Bézier curves represent discontinuity sets in piecewise constant images, often including silhouettes and pixel art [134,135]. To better fit piecewise smooth vector curves to raster boundaries, ref. [136] proposes an image vectorization method based on mathematical algorithms for frame field processing. PolyFit [137] approximates piecewise smooth vector curves to raster boundaries with coarse polygons, considering perceptual cues and simplicity. LIVE [117] and SAMVG [138] employ a layer-wise optimization framework that significantly improves vectorization quality. Chen et al. [139] explore the assembly of simple parametric primitives within a neural network for geometric abstraction. SuperSVG [140] focuses on decomposing the input into superpixels for optimized reconstruction and detail refinement.

### 4.2. Text Element Generation

#### 4.2.1. Artistic Typography Generation

One of the main directions in text element generation is font style learning. Traditional methodologies centered on explicit shape modeling and statistical learning techniques to craft font glyphs of calligraphy, predominantly targeting elements such as strokes and radicals [141,142]. Research efforts have focused on emulating traditional calligraphic styles using hierarchical models and texture transfer techniques [143,144,145,146]. Deep learning has markedly increased the flexibility and realism in font creation; researchers have utilized image translation methods for font generation and explored font style learning in one-shot and few-shot settings. Ref. [147] was the first to adopt GANs to automatically generate a Chinese font library by learning a mapping from one style font to another, and DC-Font [148] also addresses the font feature reconstruction and handwriting synthesis problems through adversarial training. However, these methods operate under supervised learning and necessitate a large volume of paired data. Some methods employ auxiliary annotations (e.g., stroke and radical decomposition) to enhance generation quality further. RDGAN [149] proposes a radical extraction module to extract rough radicals. To facilitate the automatic synthesis of new fonts more easily, some works follow unsupervised methods to separately obtain content and style features and then fuse them in a generator to produce new characters [150,151,152]. Concurrently, other works leverage auxiliary annotations to make the models cognizant of the specific structure and details of glyphs [36,38]. Most recently, Diff-Font [37] represents a pioneering effort to use a diffusion model, treating it as a conditional generation task to manage content through predefined embedding tokens while extracting the desired style from a one-shot reference image.

Another significant line is transferring artistic styles of color and texture onto new glyphs. Yang et al. [153] pioneered text effect transfer by enabling the migration of effects from a stylized text image to a plain one. Following this, ref. [154] developed a general framework for user-guided texture transfer, applicable to a variety of tasks, including transforming doodles into artworks, editing decorative patterns, generating texts with special effects, controlling effect distribution in-text images, and swapping textures. The following research in this domain revolves around exploring and enhancing techniques for separating, transforming, and recombining text styles and content. Research has progressively evolved to address various aspects of style and content encoding, mixing, and decoding. Ref. [155] pioneered the application of deep networks for text effect transfer, focusing on combining font and text effects. Ref. [156] addressed specific issues like stroke adhesion and text clarity, while [157] tackled data scarcity through synthetic data generation. The field has recently seen innovative approaches leveraging diffusion models for diverse style support and interactive generation, culminating in Wang et al. [158]’s method for generating artistic fonts using a text-to-image diffusion model.

In semantic typography, the primary goal is to enable the integration of artistic expression with legibility while embedding semantic meanings into typographic designs. Xu et al. [159] pioneered an interactive method for creating calligrams that warped letters to fit within specific regions of an image, aligning them semantically with the visual content, albeit sometimes at the cost of readability. Building on the need for clarity, Zou et al. [160] refined guidelines for glyph deformation through a crowd-sourced study, aiming to improve the readability of automated letter layouts. To further personalize and enrich typography, Zhang et al. [161] introduced a framework that allowed users to influence glyph structure interactively, incorporating a semantic-shape similarity metric and optional structural optimization techniques to enhance both aesthetics and integrity. Advancements continued with Iluz et al. [127] who modified letter geometry based on semantic meanings and employed advanced rendering techniques to ensure high-quality visualization across sizes. Finally, Tanveer et al. [162] leveraged large language models and unsupervised generative models to synthesize stylized fonts with embedded semantic meanings.

#### 4.2.2. Visual Text Rendering

Text rendering aims to render the text characters in imagery. Methodologies in this area have been extensively researched to address the issue of visual inconsistency often observable when text is merely superimposed onto images. Notable approaches include SynthText [163], VISD [164], SynthText3D [165]. Despite these technological advancements, the field continues to face significant challenges in achieving accurate text rendering and ensuring visual coherence with the surrounding environment, primarily due to the limited diversity in background datasets utilized for training and synthesis. Most existing research efforts [166] have concentrated on the precise visual rendering of text in English. However, initiatives like AnyText [167] show only moderate success in rendering texts in other languages such as Chinese, Japanese, and Korean. This is largely attributed to the challenges in gathering high-quality data and the constraints of training models on a limited dataset comprising merely 10,000 images across five languages. Given the extensive array of characters in these non-English languages, such a dataset size proves inadequate for comprehensively addressing the task of multilingual visual text rendering. Furthermore, contemporary commercial image generation models like DALL·E3, Imagen3, Stable Diffusion 3 [168], and Ideogram 1.01 have demonstrated underwhelming performance in multilingual text rendering tasks.

Recent research has focused on enhancing text rendering accuracy by integrating large-scale language models such as T5, used by platforms like Imagen [169]. Studies suggest that character-aware models like ByT5 [170] offer substantial advantages over character-blind models such as T5 and CLIP [171] in terms of text rendering accuracy. Innovations such as GlyphDraw [172] introduce frameworks for precise control over character generation, incorporating features like auxiliary text locations and glyph characteristics. TextDiffuser [166] uses a layout transformer to enhance knowledge of text arrangement and integrates character-level segmentation masks for higher accuracy. GlyphControl [173] and Diff-Text [174] refine the approach by facilitating explicit learning of text glyph features and using rendered sketch images as priors for multilingual generation, respectively. Meanwhile, GlyphOnly [175], which uses glyphs and backgrounds for accurate rendering and consistency control, is equipped with an adaptive strategy for exploring text blocks in small-scale text rendering.

### 4.3. Layout Generation

#### 4.3.1. Automatic Layout Generation

Layout can be created by selecting a template that best fits the content [176,177,178]. However, such a predefined, constrained set of templates can rarely accommodate the vast diversity of graphic design layouts. Many works have studied the creation of a layout according to the given elements for graphic design such as UI design [179], advertisement design [180,181], website [182], book covers [183], magazine design [92], and poster design [184,185], among others. Early research on design layouts primarily utilized templates, exemplars, and heuristic design rules [186,187,188,189]. These methods, which often required professional design knowledge, leveraged predefined templates or heuristic-based rules but struggled to address the diversity and complexity of design requirements effectively. Subsequent developments introduced techniques such as saliency maps [190] and attention mechanisms [191]. These methods were designed to assess the visual importance within graphic designs, track user attention, and enhance understanding of how users engage with visual elements, marking a significant step towards understanding the dynamics of visual interaction in layouts. Neural networks enable researchers to derive design principles from extensive datasets. CanvasVAE [123] introduced a VAE-based architecture for unconditionally generating vector graphic documents. Following this, LayoutGAN++ [192] further refined this approach by incorporating user-specified constraints into layout generation. LayoutDM [193] employs DDPM to handle geometric parameters in continuous spaces while introducing category information as a condition. LayoutDiffusion [194] treats both geometric parameters and category information as discrete data. LDGM [195] proposes to decouple the diffusion processes to improve the diversity of training samples and learn the reverse process jointly. These methods are learned from the vector domain. A recent work [196] combines the advantages from both bit vector and raster image spaces by proposing a dual diffusion model for design layout generation.

In addition, layout generation in raster images has evolved into two directions: content-agnostic and content-aware layout generation. Content-agnostic layout generation focuses on generating layouts without a predefined content structure. Techniques such as LayoutVAE [197], which utilizes a VAE, and others employing auto-regressive models [198,199,200] or diffusion models [201,202] have been prominent. DLT [203] further advances this by integrating discrete and continuous data in a diffusion layout transformer. Content-aware layout generation integrates specific visual and textual content into the layouts, aiming to create more contextually relevant designs. Early innovations include Content-GAN [204], which was the first to combine visual and textual elements. Subsequent models like and ICVT [205] employed transformer-based networks and conditional VAEs, respectively, to enhance content integration. PosterLayout [206] uses a CNN-LSTM network focusing on saliency maps. LayoutDETR [207] leverages a detection transformer approach, integrating GAN and VAE technologies and utilizing pre-trained visual and textual encoders for feature extraction.

Furthermore, layouts, which can be encoded in formats such as XML or JSON, are ideally suited for processing by pre-trained LLMs. To this end, a series of works utilize the paradigm of code generation + LLM [208]. LayoutGPT [3] utilizes in-context visual demonstrations in CSS structures to enhance the visual planning capabilities of GPT-3.5/4 for generating layouts from textual conditions. MuLan [209] iteratively plans the layout of an image by deconstructing the text prompt into a sequence of subtasks with an LLM, then revises the image at each step based on feedback from a vision–language model. TextLap [210] enables users to generate layout designs based on natural-language descriptions. LayoutPrompter [211] introduces a training-free approach by leveraging Retrieval-Augmented Generation to enhance the in-context learning capabilities of GPT [212], dynamically sourcing examples from a dataset. However, this retrieval-centric strategy is limited to open-domain generation. These works often overlook the visual domain features or convert them into hard tokens before inputting them into LLMs, which can result in significant information loss.

#### 4.3.2. Glyph Layout Generation

Wang et al. [213] was the first to propose this task. The synthesized layouts of glyphs must consider fine-grained details, such as avoiding the collision of strokes from different glyphs. Furthermore, the placement trajectories of characters should follow a correct reading order (e.g., left to right and top to bottom for English) and possess diverse styles simultaneously, challenges that non-sequence generation models struggle to handle. To address these issues, the authors introduced a dual-discriminator module designed to capture both the character placement trajectory and the rendered shape of the synthesized text logo. However, it faced challenges in designing layouts for long text sequences, adapting to user-defined constraints, and providing diverse layout designs due to the limited quantity of training data. In response, GLDesigner [214] proposed a vision–language model-based framework that generated content-aware text logo layouts by integrating multimodal inputs with user constraints. That study also included the creation of two extensive text logo datasets, which were five times larger than any existing public datasets. In addition to geometric annotations, such as text masks and character recognition, comprehensive layout descriptions in natural language format were provided to enhance reasoning capabilities. Although that model indeed improved the fidelity of generated visual text, it generally fell short in rendering longer textual elements. Lakhanpal et al. [215] introduced a training-free framework to enhance two-stage generation approaches focusing on generating images with long and rare text sequences.

### 4.4. Colorization

Image colorization is the process of converting grayscale images, including manga [216], line art [217], sketches [218,219], and grayscale photographs [220], into their full-color versions. Various techniques are employed to guide the colorization process, such as scribbles [221,222,223], reference images [224,225,226,227,228,229,230,231,232], color palettes [230,233], and textual descriptions [220,234,235,236]. Scribbles are used to provide intuitive and spontaneous color hints through freehand strokes. The Two-stage Sketch Colorization [237] incorporates a CNN-based system that first applies preliminary color strokes to the canvas, which are later refined to improve color accuracy and detail. Colorization using reference images involves transferring color schemes from an image with similar elements, scenes, or textures. Methods based on stroke application, or edit propagation, allow users to manually introduce color alterations using strokes that are algorithmically extended across the image based on criteria like color similarity and spatial relationships. These methods are invaluable for targeted color adjustments and preserving the authentic appearance of the image. Developments in this field have introduced neural network-driven techniques that automate edit propagation across comparable image structures [238,239]. Palette-based techniques aim to distill the essential color scheme of an image into a select group of representative colors, thereby reducing and abstracting the rich diversity of colors present. Innovations by Chang et al. involved adapting a K-means clustering algorithm to extract a color palette, which laid the groundwork for later advancements. Palette-based models [240] utilize the selected palette as a stylistic guide to influence the overall color theme of the image. Example-based approaches, or style transfer, utilize existing images as templates to guide the recoloring effort, allowing for the transfer of stylistic color elements from one image to another, a process enhanced through the use of CNNs and GANs [7,8]. With the rise of diffusion models, textual descriptions have become a pivotal tool for image generation and thus play a significant role in image colorization. Text-based guidance employs descriptions of desired color themes, object colors, or mood. ControlNet [236] integrates additional trainable modules into pre-existing text-to-image diffusion models [241], tapping into the inherent capabilities of diffusion models for colorization tasks.

### 4.5. Summary

SVGs use geometric primitives like Bézier curves, polygons, and lines, making them well suited for representing visual concepts in a structured, scalable format. DiffVG [116] allows seamless transitions between raster and vector images. Research in visual text generation can be divided into three main categories: basic text generation, artistic text generation, and text rendering in natural scene images. (1) Basic text generation primarily deals with font transfer, especially for complex scripts such as Chinese or Korean. The focus extends beyond simple generation to include text segmentation into its constituent parts like radicals and strokes, which helps guide the learning process more effectively. (2)  Artistic text generation encompasses two main tasks: artistic style transfer and semantic text generation. Both areas use the same basic generation models but tackle different challenges. Artistic style transfer focuses on separating the style and content of text. Semantic text generation, conversely, involves self-deformation of text to maintain readability and aesthetic appeal during automatic re-layout. (3) The rendering of text in natural scene images is particularly challenging due to the need for high clarity and accuracy in diverse visual contexts. To address these challenges, researchers utilize large pre-trained models like Google’s T5 series, which is adept at character perception.

The study of layout is a prominent topic. Traditional template-based methods, prevalent in early research, often struggle to encapsulate design rules effectively. Contemporary training-based approaches are categorized based on the type of data used: bitmap data and raster image data. Additionally, a recent innovative work [196] integrates a dual diffusion model encompassing bitmap and raster images. Furthermore, another significant advancement is the encoding of layouts in formats such as XML or JSON, which are highly compatible with processing by pre-trained LLMs. Recent studies have begun to conceptualize layout generation as a language reasoning or planning task. Further developments include the integration of visual information.

Aesthetic research specifically focuses on recolorization. Traditionally, coloring methods have predominantly utilized rule-based systems complemented by color propagation strategies. CNNs were trained on paired grayscale and color images, facilitating the learning of direct mappings from grayscale inputs to their colorized counterparts. Further developments saw the introduction of GANs. In this setup, a generator network learns to create color images that emulate the real colors found in the dataset, while a discriminator network ensures these colorizations are consistent with the original grayscale images. Despite the success of CNNs, their limitations in capturing long-range dependencies prompted researchers to explore other architectures. The introduction of transformers, known for their ability to handle global contexts, brought new opportunities. The most recent advancements involve diffusion models, which integrate pre-trained models to better understand the semantics of color. These models offer guidance during the colorization process by leveraging learned representations of color and its contextual significance, leading to more nuanced and semantically coherent outputs.

## 5. Present and Future

In Table 2, we summarize the comparison of technologies in AIGD. In this section, we examine the current trend of AIGD, identifies key challenges, and outline future directions and the prevailing research trend of addressing comprehensive problems through holistic solutions.

### 5.1. Analysis Within Perception Tasks

Perception tasks in AIGD focus on analyzing and interpreting design elements, forming the bedrock for intelligent design systems. Here, technologies such as object recognition using MLLMs and DETR-based detection excel in accuracy by leveraging advanced vision–language integration to precisely identify visual objects in raster images. However, when compared to SVG recognition methods (e.g., YOLaT with Graph Neural Networks), object recognition demonstrates superior adaptability in handling complex, multi-level abstractions in vector graphics, though it often incurs higher computational costs due to the semantic processing overhead, making it less efficient for real-time applications in resource-constrained design tools. In terms of usability, MLLMs enhance designer workflows by providing textual outputs that align with natural language queries, but semantic gaps limit their reliability in nuanced graphic contexts, such as interpreting abstract art.

Text element perception technologies, including OCR variants like TextBoxes [56] and Mask R-CNN [61], offer high efficiency in processing diverse scenes, with generative models supporting quick adaptations to irregular text. Yet, compared to font recognition approaches (e.g., CNNs with FontCLIP [242]), OCR suffers from lower accuracy in noisy or variable environments due to complex post-processing needs, reducing its adaptability for multilingual or stylized fonts in global design projects. Usability is a strength for font recognition, as tools like GANs enable flexible font selection with minimal user intervention, though dataset complexity poses barriers for non-expert designers.

Layout analysis methods (e.g., hybrid top-down/bottom-up with transformers) strike a balance in efficiency by managing irregular layouts through pixel-based extraction, outperforming specialized perception tools in adaptability to complex documents like infographics. However, their computational intensity hampers usability in everyday graphic design software, where faster alternatives like Faster-RCNN might be preferred despite occasional inaccuracies in table handling.

Aesthetic understanding technologies reveal stark contrasts: color palette recommendation (e.g., VAEAC [243] and transformers) provides dynamic, region-specific outputs with high efficiency for iterative design but overlooks semantics, leading to suboptimal accuracy compared to other attribute models (e.g., DMA-Net), which encode global layouts and user perceptions more adaptively. Usability favors personalized models, yet the subjective nature of aesthetics lacks standardized benchmarks, making cross-technology comparisons challenging in collaborative design environments.

Overall, perception technologies prioritize accuracy and adaptability in structured tasks but lag in efficiency for high-volume design workflows, with usability often compromised by training dependencies.

### 5.2. Analysis Within Generation Tasks

Generation tasks shift toward creating new design elements, where AI’s creative potential shines but introduces variability in performance metrics. Non-text element generation, exemplified by SVG generation (e.g., DeepSVG with VAEs and diffusion models), achieves high scalability and precise control, offering superior efficiency over vectorization techniques (e.g., PolyFit with optimization), which preserve details but struggle with boundary alignment, reducing accuracy in perceptual fidelity. Adaptability is a key differentiator: text-conditioned SVG models like VectorFusion excel in diverse graphic styles, enhancing usability for designers experimenting with AI-assisted ideation, though dataset dependencies limit their robustness in novel scenarios.

In text element generation, artistic typography (e.g., Diff-Font with GANs) provides flexibility for complex scripts, surpassing visual text rendering (e.g., TextDiffuser [244], TextDiffuser-2 [166], Artist [245]) in adaptability to multilingual designs, but at the cost of readability trade-offs that diminish accuracy. Efficiency favors diffusion-based rendering due to interactive segmentation, improving usability in real-time editing tools, yet data scarcity hampers both in underrepresented languages.

Layout generation technologies, such as automatic layouts with LayoutGAN [246] and LLMs, handle diverse requirements efficiently via natural language inputs, offering greater usability than glyph layouts (e.g., GLDesigner [214]), which provide high fidelity but falter with long texts. Accuracy in constraint handling makes glyph models more adaptable for precise typography, though visual feature loss in automatic methods reduces overall effectiveness in multilayered compositions.

Aesthetic refinement, particularly colorization (e.g., ControlNet [236] with GANs), automates adjustments with semantic awareness, excelling in efficiency and usability for guided edits, but long-range dependencies lead to inconsistencies, lowering accuracy compared to palette-based approaches in scene-specific tasks.

Generation technologies generally outperform perception ones in adaptability and usability by enabling creative outputs, yet they demand higher efficiency in handling dependencies, with accuracy often traded for speed in generative processes.

### 5.3. MLLM for Graphic Design

Across perception and generation, a clear synergy emerges: perception technologies like MLLMs feed into generation tasks (e.g., text-conditioned SVG), enhancing overall accuracy in end-to-end workflows. However, efficiency gaps persist—perception’s computational demands slow generation in integrated systems, while adaptability favors multimodal approaches that bridge visual and semantic gaps. Usability is amplified in 2025 trends like AI realism and personalization, where tools such as Runway ML automate repetitive tasks, allowing designers to focus on empathy and originality. Challenges remain in interpretability and human intent alignment, with generative AI risking over-automation that erodes designer control. In graphic design contexts, these comparisons reveal AI as an augmentative force rather than a replacement, boosting efficiency in mundane tasks while demanding human oversight for adaptability in creative, user-centric projects. Future directions should prioritize hybrid models integrating LLMs for better intent understanding, alongside ethical considerations for bias in datasets, to foster more inclusive and usable AIGD ecosystems. We provide a pseudocode example framework that handles multimodal inputs and illustrates the methodology behind integrating text and visual prompts. Pseudocode can be found in Listing 1 below.

**Listing 1.** Pseudocode for Multimodal Input Processing with Practical Integration.

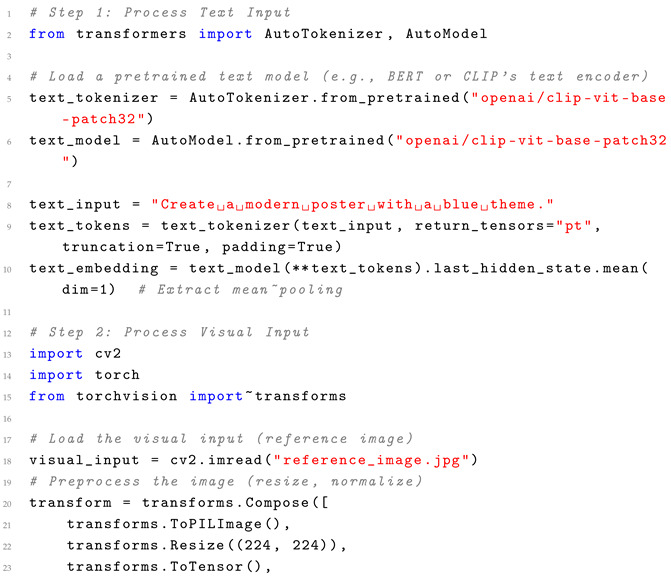



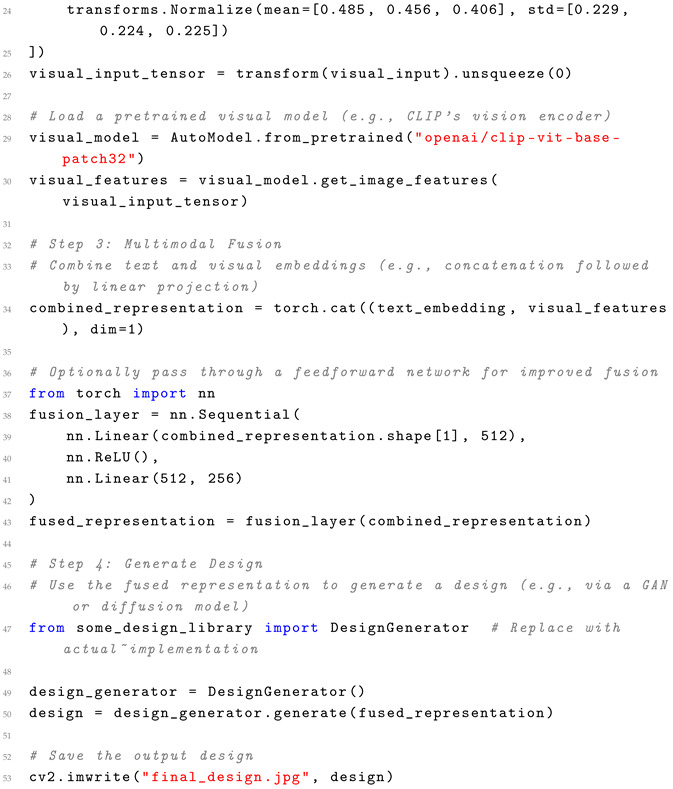



**Figure 4:**
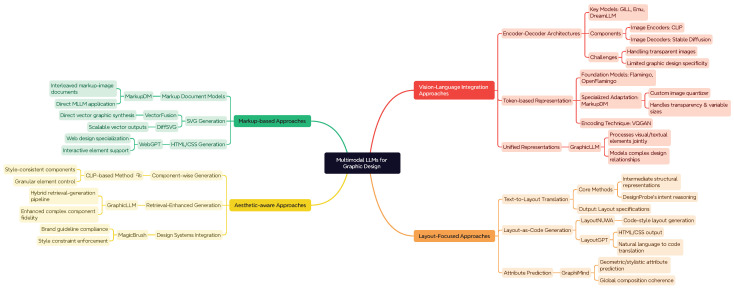
Overview of existing works in multimodal LLM for graphic design.

Recent advancements in multimodal LLMs have shown promising applications in graphic design tasks. As shown in Figure 4, these approaches can be categorized into several key directions based on their architectural design and application focus.

Vision–language integration approaches connect LLMs with pre-trained image components:

- Encoder–Decoder Architectures: Models such as DreamLLM [247] integrate LLMs with pre-trained image encoders (e.g., CLIP [171]) and decoders (e.g., Stable Diffusion [241]). While powerful for general image generation, these approaches face challenges with transparent images common in graphic design [248]. OpenCLOE [249] begins by translating user intentions into a design plan using GPT-3.5 and in-context learning. Then, the image and typography generation modules synthesize design elements according to the specified plan, and the graphic renderer assembles the final image.
- Token-based Representation: An alternative approach represents images as discrete tokens [10,250,251]. This method encodes images into token sequences via image quantizers like VQGAN [252]. The MarkupDM approach [248] adapts this methodology specifically for graphic design by developing a custom quantizer that handles transparency and varying image sizes.
- Unified Models: GraphicLLM [253] proposes a multimodal model that processes both visual and textual design elements within a unified framework, addressing the complex relationships.

Layout-focused approaches leverage LLMs specifically for layout generation tasks:

- Text-to-Layout Translation: The authors of [254] utilize LLMs to translate descriptions into intermediate structural representations that guide subsequent layout generation. DesignProbe [19] extends this by introducing a reasoning mechanism where LLMs analyze design intent before generating structured layout specifications.
- Layout-as-Code Generation: LayoutNUWA [208] treats layout generation as a code generation task, leveraging the programming capabilities of LLMs. Similarly, LayoutGPT [3] functions as a layout generator by producing HTML/CSS code from textual prompts.
- Attribute Prediction: GraphiMind [25] employs MLLMs to predict geometric and stylistic attributes for design elements while maintaining global coherence across the entire composition.

Aesthetic-aware approaches aim to address multiple aspects of design simultaneously:

- Component-wise Generation: The authors of [255] propose a method that leverages CLIP embeddings to generate design components that maintain stylistic consistency. VASCAR [256] is large vision–language model-based content-aware layout generation. Design-o-meter [257] is the first work to score and refine designs within a unified framework by adjusting the layout of design elements to achieve high aesthetic scores.
- Retrieval-Enhanced Generation: GraghicLLM [253] combines generative capabilities with retrieval mechanisms to leverage existing design elements, achieving higher fidelity results for complex graphic components.
- Design Systems Integration: MagicBrush [258] integrates with design systems to ensure generated elements conform to established brand guidelines and stylistic constraints.

Markup-based approaches involve representing designs as markup language:

- Markup Document Models: MarkupDM [248] introduces a novel approach treating graphic designs as interleaved multimodal documents consisting of markup language and images. This representation allows direct application of multimodal LLMs to graphic design tasks.
- SVG Generation: VectorFusion [126] focus on generating vector graphics (SVG) directly, addressing the scalability advantages needed for professional graphic design workflows.
- HTML/CSS Generation: WebGPT [259] generates web-based designs by producing HTML and CSS code, demonstrating the potential of code-centric approaches for interactive designs.

MLLMs such as LayoutGPT represent a significant advancement in automated layout and graphic design. However, their performance is often evaluated using a combination of general-purpose and task-specific metrics, including the following: BLEU measures the similarity of generated layouts to reference layouts, particularly in structured tasks like table or form generation; Fréchet Inception Distance (FID) evaluates the visual quality of generated outputs by comparing them to real-world designs, measuring how realistic and well aligned the results are; and Alignment Accuracy quantifies the placement of elements (e.g., text, images, buttons) with respect to design guidelines or user-defined constraints.

Traditional methods often rely on predefined templates and rules, which limit flexibility but ensure quick execution for specific tasks. In contrast, LayoutGPT leverages generative capabilities to create custom layouts dynamically, enabling scalability across various design domains. While traditional methods ensure consistent adherence to design principles, they may struggle with complex or non-standard layouts. LayoutGPT, powered by deep learning, generates visually diverse and highly creative designs but may occasionally produce outputs that require manual refinement. Multimodal LLMs like LayoutGPT excel in automating the design process, reducing manual effort through intelligent suggestions and rapid prototyping. Unlike rule-based systems, they adapt to user-specific requirements and diverse input modalities, including text, images, and sketches. A case study can be found in Listing 2 below.

**Listing 2.** Case study: traditional template-based graphic design.

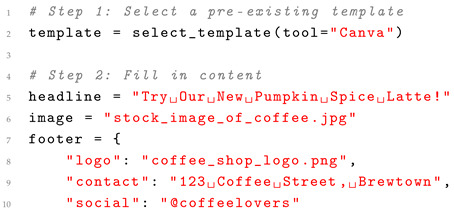



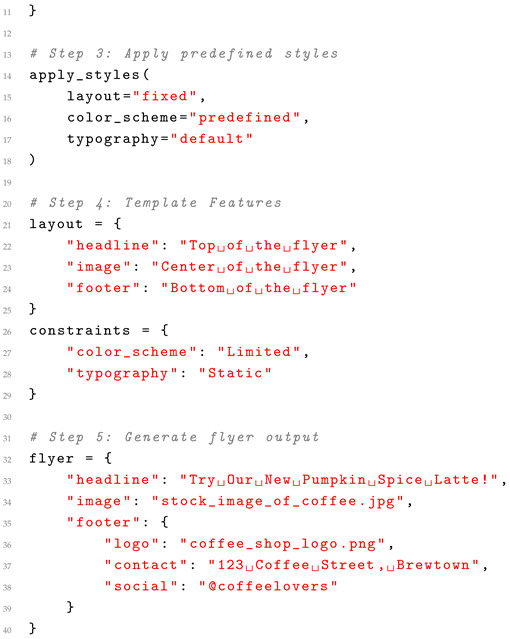



### 5.4. Existing Challenges

**Figure 5:**
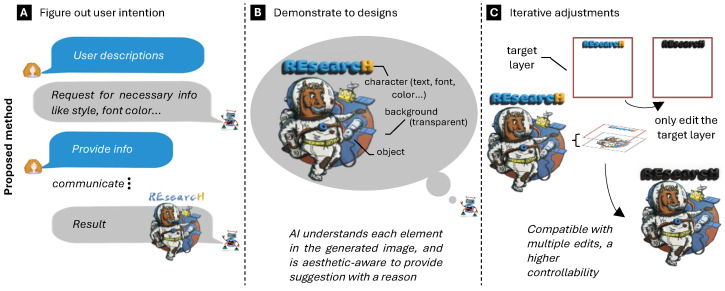
Demonstration of AIGD characteristics, including autonomy in (**A**) to figure out user intention, interpretability in (**B**) where the explanation can be provided to demonstrate designs, and multilayered aspect in (**C**) when editing generated designs.

Despite remarkable advances in AIGD, significant technical barriers that limit practical applications persist. As illustrated in Figure 5, conventional approaches suffer from three fundamental limitations: inadequate user intention understanding, limited interpretability, and insufficient layer control. These challenges reflect deeper systemic issues within AI architectures requiring targeted research attention.

The problem of inadequate user intention understanding (Figure 5A) represents a fundamental cognitive–computational gap in current systems. While text-to-image models have advanced significantly, they fundamentally operate through statistical pattern matching rather than the semantic understanding of design requirements. This semantic–representational decoupling manifests when converting textual briefs to visual styles, where the design intention encoding $hI=\mathrm{LLM}\theta \left(x\mathrm{text}\right)⊕\mathrm{ViT}\phi \left(x_{\mathrm{image}}\right)$ yields demonstrably lower style consistency than human designers in controlled trials. The challenge extends beyond simple prompt engineering to the more complex problem of modeling designers’ cognitive processes when translating abstract requirements into concrete visual decisions. Current multimodal approaches attempt to bridge this gap through joint embeddings but fail to capture the nuanced contextual reasoning that experienced designers apply to interpret client needs, audience expectations, and brand guidelines simultaneously [248,253]. This intention–representation gap becomes particularly evident in iterative feedback scenarios, where AI systems struggle to incorporate targeted revisions without regenerating entire compositions. This limitation significantly reduces their utility.

The interpretability challenge (Figure 5B) reflects a deeper epistemological problem in AI-driven design: the inability to articulate design rationale in terms that align with established design principles and practices. Current systems provide generic explanations that lack specificity regarding composition decisions, color harmony, typographic choices, and other critical design elements. This limitation stems partly from how design knowledge is encoded during training—predominantly through implicit pattern recognition rather than explicit design theory. The catastrophic performance degradation when transitioning from atomic to composite tasks represents a direct consequence of this interpretability deficit. Models cannot effectively decompose complex tasks into meaningful subproblems or explain the interrelationships between design elements without an explicit representational framework for design principles. Recent work in design rationale extraction has attempted to explain generated designs retroactively. Still, these post hoc rationalizations often fail to reflect the actual generative process or provide actionable insights for refinement. Typography presents an additional interpretability challenge—while font generation models have achieved impressive stylistic accuracy, they frequently fail to balance aesthetic considerations with functional requirements like readability across contexts, proper kerning, and linguistic nuance.

The multiple layers and iterative editing problem (Figure 5C) reveals fundamental architectural limitations in current generative models when applied to professional graphic design workflows. Unlike the photography-oriented generation, graphic design requires precise control over individual elements, relationships, and layer hierarchies. Conventional methods struggle with layer-specific editing—modifications to one element often unintentionally affect others, as evident in tools like TurboEdit and Flux.1. This limitation stems from pre-trained image encoders and decoders that inadequately support transparent images and multilayered compositions [248]. The technical challenge extends beyond simple transparency support to the more complex problem of maintaining semantic consistency across layers while enabling targeted modifications. Standard diffusion models and transformers operate on flattened representations that fail to preserve the logical independence of design elements. This represents a fundamental tension between maintaining high-fidelity visual details and producing clean, scalable vector representations—a trade-off that simultaneously impacts editability, rendering performance, and file size.

Beyond these three primary challenges, a fourth systemic limitation has emerged in recent research: contextual consistency maintenance across design artifacts. Professional graphic design rarely involves isolated images, instead requiring coherent visual systems spanning multiple formats and applications while maintaining brand identity. Current AI approaches treat each generation as an independent task, lacking the architectural components to model and maintain cross-artifact consistency. This limitation becomes particularly problematic in comprehensive design systems where visual elements must adapt to different contexts (responsive web design, print media, environmental applications) while preserving core identity elements. The computational challenge involves maintaining a persistent design representation that can flexibly adapt to new constraints without sacrificing fundamental stylistic principles—a problem that remains largely unaddressed in current research. These challenges collectively point to a need for more sophisticated architectural approaches that better align with the cognitive processes, theoretical foundations, and practical workflows of professional graphic design. While recent multimodal models show promising capabilities in specific domains, bridging the gap to comprehensive design assistance requires addressing these fundamental limitations through targeted research in representation learning, explainable generative processes, compositional reasoning, and layered editing paradigms.

### 5.5. Potential Directions

Recent trends in AI for graphic design suggest several promising research directions that warrant further investigation. This section explores potential avenues for advancing the field, focusing on developing unified approaches and addressing specific challenges in design understanding and generation.

Towards Unified End-to-End Models. Recent advances in MLLMs demonstrate the feasibility of employing unified end-to-end solutions [260] for AIGD tasks. These models would integrate multimodal intent understanding, high-quality visual element generation, and knowledge-enhanced layout reasoning within a single framework. Such integration aligns with current academic trajectories in multimodal learning and offers a promising pathway for comprehensively addressing the complex challenges of graphic design automation.

- Multimodal Intent Understanding. Current multimodal models integrating dialogue and visual recognition provide a foundation for intent understanding but require significant enhancement in several key areas: (1) Graphic design presents unique challenges with artistic images featuring diverse fonts and complex layouts that exceed the capabilities of general-purpose recognition systems. (2) Three-dimensional designs, text with special effects (overlapping, bending, distortion), and artistic typography demand specialized recognition approaches. (3) Enhanced communicative abilities in large language models are needed to translate ambiguous user inputs into coherent, actionable design specifications.
- Knowledge-Enhanced Layout Reasoning. The computational representation of abstract design principles presents significant challenges. Drawing inspiration from advanced reasoning models like OpenAI o1, research should focus on the following: (1) Encoding established design theories within computational frameworks. (2) Developing inference mechanisms that can apply these principles contextually. (3) Creating evaluation metrics that align with human aesthetic judgment. (4) Building models that can explain their layout decisions with reference to design principles.
- High-Quality Visual Element Generation. Layer diffusion techniques show promise for creating images with transparent backgrounds—a critical requirement for graphic design. However, text generation capabilities require substantial improvement, particularly for artistic typography, where models like Flux.1 demonstrate potential but insufficient fidelity. Meanwhile, LLM-guided approaches for generating vector graphics, exemplified by tools like SVGDreamer, offer precision and scalability advantages. Research should focus on enhancing text rendering and incorporating deeper reasoning about design principles. Finally, models capable of a seamless transition between raster and vector formats could revolutionize workflow efficiency by offering the advantages of both paradigms, as suggested by [196].

Research in Sub-directions. Beyond unified models, several specialized research directions show particular promise: (1) Developing encoders specifically trained on graphic design elements could substantially improve the representation of design-specific features. Unlike general-purpose visual encoders, design-specific approaches would prioritize typographical feature representation, layout structure encoding, color harmony, and palette relationships. (2) Interactive and collaborative design systems enable iterative refinement and feedback loops between the designer and AI, focusing on turn-taking mechanisms for collaborative design, interpretable design suggestions, learning from designer feedback, and preserving creative agency while enhancing productivity. (3) Design rationale understanding models that capture the underlying reasoning in design decisions, rather than just visual patterns, represent a critical frontier [19], which involves inferring design intentions from examples, reverse-engineering design decisions, representing the relationship between design goals and visual implementations, and learning from design critique and evaluation. (4) Improved mechanisms for transferring design styles between different modalities offer significant potential for design consistency and efficiency [22]. (5) The vector graphics domain, particularly SVG, remains underexplored despite its importance in graphic design. Recent work by [261] introduces Primal Visual Description (PVD), a textual representation that translates SVG into abstractions comprising primitive attributes (shape, position, measurement) and their values. Research in this domain should explore the integration of these structured representations with generative and reasoning capabilities, potentially offering precision advantages over raster-based approaches for graphic designs.

The intersection of AI and graphic design presents rich research opportunities that bridge visual generation, multimodal understanding, and design reasoning. The emerging field of multimodal LLMs for graphic design demonstrates significant promise for automating and enhancing design workflows, particularly as these models continue to improve in handling the unique characteristics of design documents, including transparency, layout constraints, and stylistic coherence. Progress will require interdisciplinary approaches to transform design workflows while preserving and enhancing human creative agency.

Potentials in Other Practical Applications. While this survey primarily focused on AIGD methods for vector graphics and typography, the methodologies discussed are highly adaptable and extendable to other domains, such as UI/UX design and print media. For example, in the domain of UI/UX design, AIGD methods such as automated layout generation have been successfully implemented to optimize interface design. Case studies illustrate how AI-driven layout tools suggest user-friendly arrangements of buttons, menus, and other UI components. These methods leverage constraints like usability heuristics and accessibility guidelines to create dynamic layouts that adapt to diverse screen sizes and user preferences. Similarly, Adobe XD’s AI-powered tools demonstrate how AI can assist designers in decision-making processes, such as selecting color schemes, typography, and interaction patterns based on user behavior data and cognitive load models. Generative design techniques, such as those employed by Figma’s Variants feature for prototyping, enable rapid iterations by producing multiple design variations for A/B testing or user feedback collection. The principles explored in this survey, particularly for typography and vector graphics, also find direct applications in print media. For instance, AI’s role in creating consistent typographic hierarchies and vector-based layouts that scale seamlessly across different print formats has been well documented. A notable case study is Canva’s AI-powered design assistant, which helps users create brochures, posters, and packaging designs that align with brand guidelines. Similarly, Coca-Cola’s use of AI-generated packaging designs highlights how AIGD can produce visually cohesive and on-brand materials for mass production. Furthermore, AI-driven tools like Adobe InDesign’s Liquid Layouts have enabled the creation of personalized print materials, such as tailored advertisements or invitations, by combining user data with generative design techniques. The underlying algorithms for layout generation, typography optimization, and style synthesis are domain-agnostic, as demonstrated in studies like Microsoft’s Project Trove, which facilitates seamless transitions between digital (UI/UX) and physical (print) design contexts. By incorporating domain-specific constraints—such as print resolution for physical media or screen responsiveness for digital outputs—AIGD methods have been shown to support diverse design workflows across industries.

## 6. Conclusions

This survey comprehensively reviewed AI’s state-of-the-art methods and applications in graphic design, categorizing them into perception and generation tasks. We explored various subtasks, including non-text element perception, text element perception, layout analysis, aesthetic understanding, and the generation of non-text elements, text elements, layouts, and colors. Integrating large language models and multimodal approaches has become a pivotal trend, enabling more holistic and context-aware design solutions. However, several challenges persist, such as the need to better understand human intent, improved interpretability of AI-generated designs, and enhanced control over multilayered compositions. Future research should develop unified end-to-end models integrating multimodal understanding, high-fidelity generation, and design reasoning. 

## Figures and Tables

**Table 1:** Comparison of surveys in graphic design intelligence with quantitative metrics.

Surveys	Focus Area	Key Contribution	Papers Reviewed
Tian et al. (2022) [13]	Mathematical foundations and content creation stages of vector graphics	Provides a foundation for understanding vector graphic representation and its mathematical principles	147
Shi et al. (2023) [14]	Layout generation aesthetics and technologies	Explores technologies for automatic layout generation, focusing on aesthetic rules	131
Huang et al. (2023) [9]	Taxonomy of graphic design intelligence	Categorizes approaches to graphic design tasks into a taxonomy of AI-driven intelligence	77
Liu et al. (2024) [15]	Graphic layout generation: implementation and interactivity	Reviews techniques for interactive and automated layout generation	40
Tang et al. (2024) [16]	Challenges and future needs for AI tools in graphic design	Identifies challenges and future functional needs based on designer interviews	39
Ours	Unification of cognitive and generative tasks in design workflows	Proposes a framework that integrates cognitive (e.g., reasoning, decision-making) and generative (e.g., image and layout generation) tasks, highlighting the interplay and potential synergies of AI-driven tools in holistic design processes	267

**Table 2:** Comparison of technologies in AIGD.

Technology	Key Features	Strengths	Limitations
Object Recognition	MLLMs, DETR-based detection	High accuracy; text integration	Limited coverage; semantic gaps
SVG Recognition	Graph-matching; YOLaT, GNNs	Effective for vectors; abstraction	Ignores high-level info; GNN limits
OCR	TextBoxes, Mask R-CNN, DETR	Diverse scenes; generative support	Complex post-processing; training issues
Font Recognition	CNNs, SVMs, FontCLIP, GANs	High accuracy; flexible fonts	Font variability; dataset complexity
Layout Analysis	Top-down, bottom-up, hybrid; transformers	Handles irregular layouts; pixel-based	Computationally heavy; table issues
Color Palettes	Regression, VAEAC, transformers	Dynamic, region-specific	Semantic oversight; suboptimal predictions
Other Attributes	CNNs, DMA-Net; personalized models	Global layout encoding; user-focused	Subjective; lacks benchmarks
SVG Generation	SketchRNN, DeepSVG, VectorFusion; VAEs	Scalable; precise; text-conditioned	Dataset dependency; complex synthesis
Vectorization	PolyFit, LIVE, SAMVG; optimization	Preserves detail; high quality	Boundary alignment; perceptual issues
Artistic Typography	DC-Font, Diff-Font; GANs, diffusion	Flexible; supports complex scripts	Data scarcity; readability trade-offs
Visual Text Rendering	TextDiffuser, GlyphControl; multilingual	Accurate segmentation; interactive	Limited dataset diversity; clarity issues
Automatic Layout	LayoutGAN, LayoutGPT; VAEs, LLMs	Diverse needs; language-driven	Complex layouts; visual feature loss
Glyph Layout	GLDesigner; vision–language models	High fidelity; constraint handling	Long text issues; limited data
Colorization	ControlNet, Palette-based; CNNs, GANs	Automated; semantic-aware	Long-range dependencies; inconsistencies

## Data Availability

No new data were created or analyzed in this study.
